# Health Impact Assessment of Urban Development Project

**DOI:** 10.5539/gjhs.v8n9p224

**Published:** 2016-01-31

**Authors:** Parisa Shojaei, Masoud Karimlou, Farahnaz Mohammadi, Hosein Malekafzali

**Affiliations:** 1 Social Determinants of Health Research Center, Welfare and Rehabilitation University of Medical Science, Tehran, Iran; 2 School of Public Health, Tehran University of Medical Sciences, Tehran, Iran

**Keywords:** Health impact assessment, Analytic Hierarchy Process (AHP), Technique for Order Preference by Similarity to Ideal Solution (TOPSIS), urban, man made lake

## Abstract

**Background:**

Health impact assessment (HIA) has emerged to identify those activities and policies likely to have major impacts on the health of a population.

**Method:**

In this research, qualitative method was applied to identifying health determinants that urban man made lake affect on them, formatting and weighing the hierarchy of the factors, calculating AHP, and Technique for Order Preference by Similarity to Ideal Solution (TOPSIS) method for decide and ranking alternatives.

**Results:**

According to the results of the study, from the structural determinants point of view, the most positive effect of man-made lake was on Recreational services by 89.5% and the most negative one was on housing. According to intermediary determinants and general average, the most positive effect of lake was on physical activity and quality of air by 88.9% and the most negative one was on noise pollution by 46.7%. Ultimately, considering the positive and negative effects of lake between constructing and not constructing the lake option, the construction option was selected.

**Conclusion:**

There is substantial potential to improve public health by bringing decision makers’ attention to the health consequences of their actions; city councilpersons, zoning commissioners, and other decision makers typically have little background in health.

## 1. Introduction

It has been proved that environmental and social factors such as community design, land use, transportation systems and so on, have a great impact on public health, but there are no sufficient sophisticated health officials or urban planners who have been trained in this field (Azapagic et al., 2013). Generally a health impact assessment (HIA) is defined as “a combination of procedures, methods, and tools by which a policy, program, or project may be judged in terms of its potential effects on the health of a population, and the distribution of those effects within the population” (Ardell, 1969; Galea, 2002; Dannenberg et al., 2006). Although social determinants and multilevel perspectives are not exclusively urban, they clearly define urban characteristics of cities such as size, density, diversity, and complexity (Vlahov et al., 2007).

The objectives of constructing Man-made Lakes can be noted: hydropower generation, improvement of navigation, flood control, irrigation, recreation, and fish culture (Brown, & Deom, 1973). According to Scotland definition, Man-made Lakes and their surroundings are parts of green spaces and open space reservation (2008). It has been proved that, green spaces increase health promotion by encouraging physical activity and walking, reducing air and noise pollution and increasing social contact (Kihal-Talantikite et al., 2013). In world, several studies have been conducted with the main focus on evaluation of health effects of urban projects, but few of them are area of Man-made Lakes. Development-projects in Iran are not health-oriented. In other words, in national development plans health promotion is treated as a side subject like other economic, cultural, social indicators and so on. According to Ministry of Health and Medical Education mission to health promotion and promoting health equity (which is the main priority in Islamic Republic of Iran’s health system), and also approval of the seventh session of the Supreme Council of Health and Food security of the country and paragraph B of 36th clause of the Fifth Economic, Social and Cultural Development Plan of Iran, is charged to prepare national standard for large developments projects (Biffi et al., 2011). Constructing Man-made Lakes faces with many hazards in developing countries. In order to exploit the full potential of water resources and lakes, improvement and maintenance of their is needed to participation of all stakeholders. Therefore in addition to engineers, the participation of other health experts particularly biology, sociology and economy in the process of planning and implementation of development projects should be considered (Araoye, 2002).

One of these projects in Iran is to construct the biggest Man-made Lake. Chitgar Lake is a Man-made Lake located in Northwest, 22 of municipality of Tehran. The lake has an area of 130 acres and there is a land area of 120 acres recreational service in the vicinity of it. The south of the lake is located in the residential area of 22 of municipality of Tehran (2013). This lake consists of three islands, green spaces, Recreational spaces, sport and games. Although the main purpose of constructing this lake is to enhance the capacity of recreation and also tourist attraction, paying no attention to the health issues cause serious problems. Because of the reasons mentioned and the need to health assessment of urban projects, we decided to assess health impact of this man made lake that the early stage of its construction was done.

## 2. Methods

To conduct the appraisal phase, 4 steps were run. In step A, a Qualitative method for Extracting health determinants associated with urban Man-made Lakes is employed, step B is weighting and prioritizing of its determinants, step C ranks the alternatives by TOPSIS, and step D Combine AHP and TOPSIS to determine the rank of alternatives.

### 2.1 A-Qualitative Method

This qualitative study was designed and carried out with a content analysis approach. Data were collected through focus group discussions (FGDs) as well as individual interviews from September to March 2013 respectively. The focus group discussions were carried out with 2 expert group in 2 session: 1- Members from the Municipal District 22 that involved in the man made lake project with at least a bachelor’s degree and 2- second group Members from outside the municipal District 22 and experts in the field of health determinants and environment to avoid information bias with at least a bachelor’s degree.

The focus group discussion was selected because this method has high ability to explore people’s ideas, worries, attitudes and experiences of individuals regarding a specific subject matter (Barbour, & Kitzinger, 1998; Dahlgren, Emmelin et al., 2007). Individual interviews were done on 34 informants in three groups: 1- people living in the 22 District, 2-Members from the Municipal District 22 and 3-third group Members from outside the municipal District 22 Informants were selected using purposive sampling and data gathering process was continued until data saturation.

### 2.2 Limitations

The present study explains the opinion of the individuals in FGDs and individual interviews and for methodological reasons (qualitative approach) the results of this study can not be generalized to other situations.

### 2.3 B-Using AHP to Analyze Priorities

In this step, after analyzing content analysis methodology, a list of all health determinants associated with Man-made Lake was extracted out by the use of Open Code software. AHP is a multi-criteria decision-making (MCDM) methodology which was developed in 1970 decade by Thomas Saaty. Analyzing complex decisions is the main usage of it which helps decision-makers to prioritize alternatives and determine the optimal alternative using pair-wise comparison judgments. Moreover, to avoid bias decision-making and provide impartiality weighting criteria by multiple experts is undertaken (Dağdeviren et al., 2009). Nevertheless, how the problem is conceived and then modeled or structured is the most important part of decision making which has a considerable effect on the outcome. Prioritization includes eliciting judgments on response to question about the dominance of one element over another when compared with respect to a specific criterion or property (Tzeng, & Huang, 2011). The philosophy behind this methodology is that, indeed a judgment is developed using numerical comparisons between two elements (or inputs) of the model which have a common criterion. A square matrix is applied to represent the set of all such judgments, in which all elements are compared with themselves. Each judgment shows the dominance of an element in the criterion list (Greenspan, 2006). It is also possible to run a Pair-wise comparison by assigning an integer ranging from 1 to 9 or the mutual of such an integer to each cell of the matrix to calculate the relative importance of the factors that characterize the cell (Saaty, 1980). By the use of this scaling approach, the available values for the pair-wise comparisons belong to the following set of numbers: (9, 8, 7, 6, 5, 4, 3, 2, 1, 1/2, 1/3, 1/4, 1/5, 1/6, 1/7, 1/8, 1/9) (Saaty, 1980). In addition, according to the assumption of AHP method, each of the factors under assessment should be independent of another.

Firstly, to execute hierarchical analysis a hierarchical tree with its aim, criterion, sub-criterion, and options was formed, and then in order to weigh all determinants extracted from qualitative method, they were put in a checklist in the form of a pair-wise comparisons matrix. In this checklist that was designed in the form of a matrix, rows and columns consist of 28 determinants (Income, Physical and Social Development of Children, Culture, Physical Activity, Education, Quality of Life, Job, Nutrition, Limitation of available resources, Unhealthy Behavior, Traffic, Unintentional Injuries, Ethnicity, Pathogenesis, Tourism, Air Quality, Region Popularity, Mental Health, Recreational Services, Aesthetic, political participation, Social Capital, Spirituality, Weather Changes, Housing, Noise Pollution, Social Security, Environmental Health) that were extracted by qualitative method. In the next step, run a Pair-wise comparison by assigning an integer ranging from 1 to 9 or the mutual of such an integer to each cell of the matrix to calculate the relative importance of the factors using Saaty method. So weights were assigned to each of them by the same participants who have participated in the interviews (experts of second group and third group). After completing checklists, by the use of geometric mean comparative tables of each respondent were jointed together. The main reason for using geometric mean is that, data created by pair-wise scales are in the form of ratio. Since the interview subjects which completed this matrix were two groups of experts, therefore after completing paired matrix, a geometric mean was computed for each group, and then by a team of experts on health determinants this geometric mean of two groups were evaluated. Finally, the review panel formed a matrix showed the overall mean, and the matrix for analysis and measurement of weights as raw data which was used into the Expert choice software.

### 2.4 C- Using Technique for Order Preference by Similarity to Ideal Solution (TOPSIS) to Rank the Alternatives

Technique for Order Preference by Similarity to Ideal Solution (TOPSIS) was represented by Yoon (1980) and Hwang and Yoon (1981), in order to solve multiple criteria decision-making (MCDM) problems based on the concept of Euclidian distance. In other words, it states that chosen alternative should have the shortest Euclidian distance from the positive ideal solution (PIS) and the farthest from the negative ideal solution (NIS) (Hwang and Yoon). This MCDM method is applied in different fields, particularly financial performance evaluation, location selection, company evaluation, supplier selection, tourism destination evaluation, selecting the most suitable machine, ranking the carrier alternatives. One of the main advantages of TOPSIS is that pair-wise comparisons are avoided (Tsaur 2011). The TOPSIS method is conducted in six steps as follows:

Step 1: Normalized decision matrix was computed. The normalized value r_ij_ is calculated as follows:


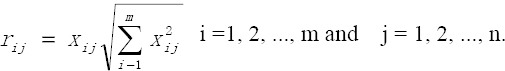


Step 2: Weighted normalized decision matrix was computed. The weighted normalized value v_ij_ is calculated as follows:


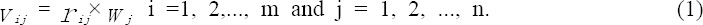


Where w_i_ is the weight of the j^th^ attribute or criterion and 
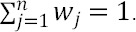
.

Step 3: the ideal (A*) and negative ideal (A^-^) solutions were determined.


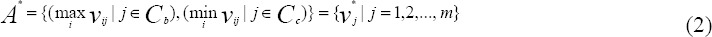



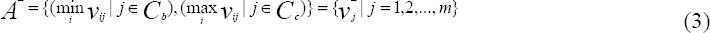


Step3: the separation measures using the m-dimensional Euclidean distance for each alternative from the positive ideal solution and the negative ideal solution were computed, respectively as follows:


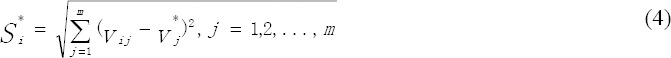



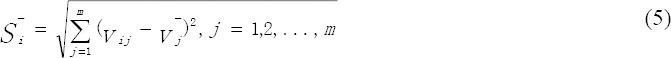


Step 5: the relative closeness to the ideal solution was computed. The relative closeness of the alternative (A_i_) with respect to (A*) is defined as follows:


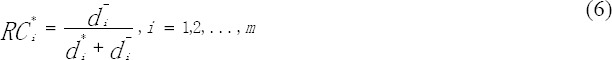


Step 6: Rank the preference order (Wu and Chuang 2013).

D- Combining AHP and TOPSIS to determine the rank of alternatives

**Figure 1:**
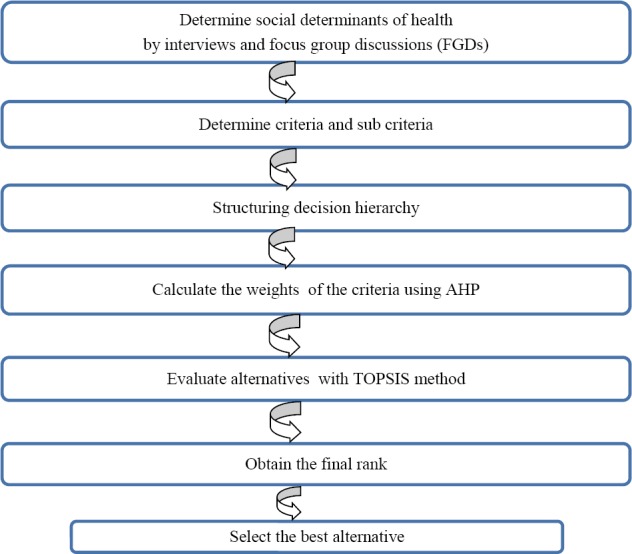
Steps of proposed method

In order to analyze the data, AHP and TOPSIS methodologies were conducted to rank alternatives. Fig 1 illustrates the steps of the proposed methods.

## 3. Results

The present study was conducted in district 22 of Tehran municipality in 2013. Participants’ mean age was obtained 30, they has been chosen among experts in urban development, environmental, water source, civil engineering, sociology, urbanization, and health determinants areas. Their education status was from BS to PhD, and also four of them were resident of the region and the others were from other parts of the city. Applying Qualitative analysis of data, 301 codes have been provided. Moreover, conceptual framework structure developed by commission on social determinants of health as well as their subgroup was treated as data analysis matrix (Solar, & Irwin, 2010). Therefore two “structural” and “intermediary” groups were created, and in each stratum14 sub-groups were determined (Table 1). Then, in order to implement AHP process these 28 determinants were put in a checklist and were given to participants through focus group discussion and interviews. Thus, there was one goal, two criteria, and 28 sub-criteria with the addition of two options (construction and non-construction of Man-made Lake) to illustrate their hierarchies.

**Table 1:** Structural and intermediary determinants of health associated with man made lake

**Structural Determinants**	Income	**Intermediary determinants**	Physical and Social Development of Children
	Culture		Physical Activity
	Education		Quality of Life
	Job		Nutrition
	Limitation of available resources		Unhealthy Behavior
	Traffic		Unintentional Injuries
	Ethnicity		Pathogenesis
	Tourism		Air Quality
	Region Popularity		Mental Health
	Recreational Services		Aesthetic
	political participation		Social Capital
	Spirituality		Weather Changes
	Housing		Noise Pollution
	Social Security		Environmental Health

According to AHP approach and with regard to structural determinants criteria, for construction option maximum final weight (.895) belonged to recreational service sub-criteria and minimum weight (.638) for traffic. These sub-criteria for non-construction option were obtained maximum (.362) and minimum (.105), respectively.

For intermediary determinants in construction option, physical activity and air quality sub-criteria had maximum final weight (.889), and also pathogenesis sub-criterion had minimum final weight (.617). These sub-criteria for non-construction option were obtained maximum (.375) and minimum (.111), respectively.

Finally, to rank the alternative suppliers TOPSIS method was conducted. Moreover, the priority weights of alternative suppliers with respect to criteria, computed by AHP, can be applied as input of TOPSIS (Table 2). The weighted normalized decision matrix was also depicted in Table 3.

**Table 2:** Input values of the TOPSIS analysis

Weight		Construction	Non Construction
0.067	Spirituality	0.86	0.14
101	political participation	0.863	0.132
0.023	Social Security	0.681	0.319
0.351	Tourism	0.889	0.111
0.186	Recreational Services	0.895*	0.105
0.245	Region Popularity	0.891	0.109
0.027	Traffic	0.638*	0.362
0.027	Ethnicity	0.804	0.196
0.028	Housing	0.667	0.333
0.085	Job	0.833	0.167
0.486	Income	0.889	0.111
0.146	Education	0.873	0.127
0.183	Culture	0.857	0.143
0.045	Limitation of available resources	0.75	0.25
0.028	Unintentional Injuries	0.786	0.214
0.317	Physical and Social Development of Children	0.877	0.123
0.069	Nutrition	0.783	0.217
0.055	Unhealthy Behavior	0.75	0.25
0.201	Quality of Life	0.857	0.143
0.304	Physical Activity	0.889*	0.111
0.027	Pathogenesis	0.617*	0.333
0.219	Mental Health	0.896	0.131
0.126	Aesthetic	0.857	0.143
0.114	Social Capital	0.872	0.128
0.029	Noise Pollution	0.667	0.333
0.107	Weather Changes	0.836	0.164
0.381	Air Quality	0.889*	0.111
0.025	Environmental Health	0.625	0.375

**Table 3:** Weighted evaluation for the supplier selection

	Construction	Non Construction		A*	A-
Spirituality	0.056	0.001	+	0.056	0.001
political participation	0.086	0.001	+	0.086	0.001
Social Security	0.014	0.003	+	0.014	0.003
Tourism	0.309	0.004	+	0.309	0.004
Recreational Services	0.165	0.002	+	0.165	0.002
Region Popularity	0.216	0.003	+	0.216	0.003
Traffic	0.014	0.004	-	0.004	0.014
Ethnicity	0.021	0.001	+	0.021	0.001
Housing	0.016	0.004	-	0.004	0.016
Job	0.069	0.001	+	0.069	0.001
Income	0.429	0.008	+	0.429	0.008
Education	0.126	0.002	+	0.126	0.002
Culture	0.154	0.004	+	0.154	0.004
Limitation of available resources	0.032	0.003	-	0.003	0.032
Unintentional Injuries Development of Children	0.021	0.001	-	0.001	0.021
Physical and Social Development of Children	0.275	0.005	+	0.275	0.005
Nutrition	0.057	0.004	+	0.057	0.004
Unhealthy Behavior	0.039	0.004	-	0.004	0.039
Quality of Life	0.17	0.004	+	0.17	0.004
Physical Activity	0.268	0.003	+	0.268	0.003
Pathogenesis	0.014	0.004	-	0.004	0.014
Mental Health	0.194	0.003	+	0.194	0.003
Aesthetic	0.106	0.002	+	0.106	0.002
Social Capital	0.098	0.002	+	0.098	0.002
Noise Pollution	0.017	0.004	-	0.004	0.017
Weather Changes	0.087	0.003	+	0.087	0.003
Air Quality	0.336	0.004	+	0.336	0.004
Environmental Health	0.013	0.004	+	0.013	0.004

As it is illustrated in Table 4, the ranking of the alternatives is construction and non-construction depends on the RCj values.

**Table 4:** TOPSIS results

Alternatives	di+	di-	RCi
Construction	0.054	0.861	0.940
Non construction	0.861	0.054	0.059

## 4. Discussion

There are many studies in the area of health impact assessment in different countries that have used quantitative and qualitative methods. Previous studies conducted with qualitative methods have used more of the interview and focus group discussion (Peters, 2002; Child Health Impact Assessment Working Group, 2005; Child Health Impact Assessment Working Group, 2006; Haigh and Scott-Samuel, 2006; University of California Berkeley Health Impact Group, 2007; Bhatia, 2010; Alameda County Public Health Department, 2013; Health Promotion Agency, 2013; Florida institute for health innovation, 2014). Qualitative studies can provide new theories about health that had not been studied about them. When stakeholders are offered in a participatory fashion, qualitative data collection analysis can be strong experiences for them. As one of the authors “most HIAs characterized impacts qualitatively; the use of quantitative analysis are deficient” (Wright, 2003; Avey, 2015).

In this study it has been showed that, constructing Man-made Lakes has significant effects on health determinants such as job, housing, income, recreational services and so on, which maximum weight belonged to recreational services, physical activity and air quality. Minimum weight also belonged to traffic and pathogenesis. Our results proved that, this Man-made Lake and its surrounding Green Space enhance the possibility of physical activity and entertainment.

Water bodies have been applied widely in urban design i.e. as decorative element, space defining element, and also as a temperature-modifying element (Lang, 1994). Singh and Bhatnagar stated that Lakes have a significant portion of water in a pristine landscape where one goes for recreation. They are the sense of nature by providing outdoor activities such as boating, camping, fishing, swimming, bird watching, and etc. However, urban lakes demystified this picture. Mostly the main purpose of constructing urban parks is to facilitate physical activity which is particularly vital in urbanized countries (Brown et al., 2014). Although urban lakes are different from the common perception of lakes in general, they have both ecosystem functions and social values as well. They generate a wide range of ecosystem services, including, air filtration, noise reduction, micro climate regulation, rain water drainage, sewage treatment, recreational and cultural values (Singh, & Bhatnagar, 2012). According to the findings, constructing lakes and their surrounding green space cause traffic there, since they appear as tourist distinctions. Results from researches of South Carolina Institute of Medicine and Public Health (2013) illustrated that the probability of heavy traffic and therefore motor vehicles accidents increase drastically in the vicinity of parks. In addition, children and elderly are the most likely group to be involved in pedestrian related accidents (2013). Therefore, alongside aesthetic and recreational values which green spaces provide and make it of great of great concern to the community, urban mangers should be aware of problems following complaints from surrounding residents (Woodward, 2008). Diseases transmit through the water, due to the low hygiene, are another disadvantage of these facilities which cause a real concern as well. Results from Brown and Deom showed that, the prevalence of the infection is increased by the stagnant water in man-made lakes. Irrigation systems are also known as areas of Schistosomiasis in so far as they increase the availability of water and consequent human contact with it; it is now proved that the man-made lakes themselves produce the same deleterious effects (Brown, & Deom, 1973). Also in Iran, Swim in stagnant water such as a lake or marsh that the flow of water in it is not fast, Can be fertile ground for the growth of microbes, parasites and are thus susceptible to various diseases such as cerebral infection (2013). Therefore, since the advantages and disadvantages of Man-made Lakes on public health, their management needs cooperation with different organizations. Although the ecological and epidemiologic aspects of Man-made Lakes have been taken into account, the application and development of a health service structure to deal with present and prospective health hazards have been underestimated. Therefore, a responsible health institution, specifically a member of health ministry, should be established to deal with the area concerned from the inception of the project involving a man-made lake since the normal authority (the health ministry) has sometimes relinquished its duties when the responsibility for health along with other concerns of the project was placed under an ad hoc institution (the dam authority or valley authority). To make a comprehensive diagnosis of the situation and establish base lines, including a complete inventory of the existing health facilities, a public health administrator planner, assisted by a group of experts in the environmental fields of epidemiology, ecology, biology, sanitation, and any others that are required (Brown, & Deom, 1973). Woodward in a study on a Man-made Lake in a metropolis of Scotland found that, approximately 80% of constructed lakes studied have experienced or are experiencing management problems along with eutrophication issues. One or more of them were reported in half (23 of the 46) lakes (Woodward 2008). One of the main issues that urban designers face in making politically and economically astute proposals for projects is that, serious environmental problems accrue in designing to meet people’s sociogenic needs. To keep the balance between the attainment of one goal and another in a way that not only is no further biogenic harm done but also future developments attain, guidelines and management manuals has been established that will shape environmental change (Majizat et al., n.d.).

To the inclusion of health in the decision-making process HIA approach can provide guidance for community leaders in the absence of good data on the effects of Man-made Lake on their citizenry health. To stakeholder involvement and acceptance of the final product, adherence to a transparent set of goals and objectives and an open and clear information-gathering and analysis process are vital (Borwick, & Pencharz, 2013). Moreover, HIA findings should be represented to decision- makers in concise, synthesized information to reach to a significant effect. Other interested audiences also include community members, journalists, advocacy organizations, and public health professionals.

As a conclusion, Due to the fact that urban populations need a different health profile and also the urban environment is substantially different from sub-urban or rural ones, urban health interventions differ from interventions in other settings. In addition, since decision-makers such as city council persons, zoning commissioners and so forth, typically have little background in health, substantial improvements occur in public health by training and bringing their attention to the health consequences of their actions (Maas et al., 2006).
